# Different papillomaviruses have different repertoires of transcription factor binding sites: convergence and divergence in the upstream regulatory region

**DOI:** 10.1186/1471-2148-6-20

**Published:** 2006-03-09

**Authors:** Santiago García-Vallvé, José R Iglesias-Rozas, Ángel Alonso, Ignacio G Bravo

**Affiliations:** 1 Evolutionary Genomics Group. Biochemistry and Biotechnology Department. Rovira i Virgili University (URV), c/Marcel-li Domingo, s/n. Campus Sescelades, 43007, Tarragona, Spain; 2 Klinikum Stuttgart. Katharinenhospital, Institut für Pathologie (Neuropathologie). Kriegsbergstr. 60, D-70174 Stuttgart, Germany; 3 Deutsches Krebsforschungszentrum. F050. Im Neuenheimer Feld-242. 69120 Heidelberg. Germany

## Abstract

**Background:**

Papillomaviruses (PVs) infect stratified squamous epithelia in warm-blooded vertebrates and have undergone a complex evolutionary process. The control of the expression of the early ORFs in PVs depends on the binding of cellular and viral transcription factors to the upstream regulatory region (URR) of the virus. It is believed that there is a core of transcription factor binding sites (TFBS) common to all PVs, with additional individual differences, although most of the available information focuses only on a handful of viruses.

**Results:**

We have studied the URR of sixty-one PVs, covering twenty different hosts. We have predicted the TFBS present in the URR and analysed these results by principal component analysis and genetic algorithms. The number and nature of TFBS in the URR might be much broader than thus far described, and different PVs have different repertoires of TFBS.

**Conclusion:**

There are common fingerprints in the URR in PVs that infect primates, although the ancestors of these viruses diverged a long time ago. Additionally, there are obvious differences between the URR of alpha and beta PVs, despite these PVs infect similar histological cell types in the same host, *i.e*. human. A thorough analysis of the TFBS in the URR might provide crucial information about the differential biology of cancer-associated PVs.

## Background

*Papillomaviridae *are a family of small dsDNA viruses that infect warm-blooded vertebrates [1]. Papillomaviruses (PVs) infect different species of mammals in the orders Primates (human, chimpanzee, bonobo, gorilla, macaque monkey, colobus monkey, and spider monkey), Carnivora (cat and dog), Perissodactyla (horse), Artiodactyla (cattle, sheep, deer, reindeer, elk), Cetacea (porpoise), Lagomorpha (rabbit and cottontail rabbit), Sirenia (manatee) and Rodentia (african soft-furred rat, hamster, porcupine). They also infect other distantly related hosts, such as marsupials (opossum) and birds (chaffinch and parrot), and it can be easily inferred that similar viruses will be found in many, if not all, vertebrates [2]. Despite the obvious absence of a sexual link in the PV cycle to ensure cohesiveness of the genome, stable PV types are identified, reflecting a sustained molecular selection through continuity in the ecological conditions and in the virus-host interactions.

Members of *Papillomaviridae *are associated to virtually all clinical cases of cervical cancer [3,4]. Others are also involved in different benign and malignant proliferative disorders, such as skin warts, genital warts, laryngeal papillomas and possibly non-melanoma skin cancer [3,5-7]. In all cases, independently of the host and the clinical manifestations, PVs infect stratified squamous epithelia, both mucosal and cutaneous. The target cells are located in the basal cell layer of the epithelia, which are available for infection *via *microlesions. The viral life cycle depends on keratinocyte differentiation. Thus, viral genomes are primarily present as nuclear episomes, which replicate in parallel to cell division. As the daughter cell migrates upwards and undergoes differentiation, the viral DNA is amplified, the viral expression pattern is modified and results finally in virion release [8].

PVs are non-enveloped viruses, with a circular dsDNA genome of ca. 8 Kb. The elements that regulate the expression of the early genes are located in the upstream regulatory region (URR) of the genome. This is a ca. 800 bp DNA stretch, spanning the region between the L1 and the E6 genes. Some of the transcription factor binding sites (TFBS) in the URR are present in all PVs, such as the binding sites for AP1, E2, NF1, Oct1, Sp1 or YY1 [9-17]. The presence of other TFBS is type-specific [18], and could therefore partially account for the differential anatomical tropism of the individual viral genotype [8]. Moreover, point mutations in the URR in variants of the same PV type lead to changes in the replication behaviour and in the transforming capacity of the viruses [19,20]. However, most of the available experimental information about the presence of TFBS refers to merely a handful of viruses, mainly classified as high-risk viruses regarding their association with cervical cancer. This is the case for HPV16, HPV18 or HPV31. For most PVs, however, the only available data concerns their genomic sequence and the epidemiological results linking them to various diseases. Their molecular characteristics, genetic maps, transcription patterns, life cycle, interaction partners and modes of action are often inferred by homology with those of the known viruses. This might have lead to an over-generalisation of the PV biology, systematically overlooking the fact that different PV types within a given genus are not genetically homogeneous, and that different PVs infecting the same host -i.e. *Alphapapillomaviruses *and *Betapapillomaviruses*- might indeed be different organisms with different biologies [21]. This is likely to be the case for the different and distantly related human PVs. In this sense systematic attempts to provide a comprehensive picture of the PVs are available [2,21], but they still do not have noticeable impact in either clinical or basic research [22].

In this paper we have addressed the *in silico *analysis of the URR of the PVs. The currently assumed hypothesis states that most of the TFBS in the URR are common to all PVs, with differences that could influence the different individual behaviours [23]. Our results suggest that the differences within and between genera are more dramatic than expected. The repertoires of TFBS present in the URR are both type- and genus-specific.

The history of the URRs in the PVs exemplifies both divergent and convergent evolution. Thus, there is an obvious divergence between different groups that once shared a common ancestor, such as delta and beta+gamma PVs. In this sense we also show that PVs that infect the same host do not necessarily share the same TFBS in the URR, as is the case in alpha and beta PVs. Finally, the presence of TFBS in the URR also illustrates convergent evolution between PVs only vaguely related but that infect related hosts. This is the case in alpha, beta, gamma, delta, mu and nu PVs. The last common ancestor of these genera, if any, is remote but they all infect primates and cluster together regarding to the repertoire of TFBS present in their URR.

## Results and discussion

### The untranslated regulatory region of the papillomavirus genome does not allow a proper phylogenetic reconstruction

We have performed a phylogenetic reconstruction of the URRs extracted from a selection of sixty-one phylogenetically representative PVs. In order to place this in a genomic context, the L1, E1 and E7 genes were also analysed. Phylogeny was reconstructed using three different alignment algorithms, four different phylogenetic algorithms, and two different nucleotide substitution models were used with each phylogenetic algorithm. Clusters were contrasted with the PV classification as revisited by de Villiers *et al*. [2]. The results for L1, E7 and the URR are given in Table 1.

**Table 1:** Phylogenetic coherence of different papillomavirus taxa according to the L1 and E7 genes, and to the URR.

L1	Clustalw				Dialign				Tcoffee				consensus
	dnapars	Fitch	NJ	UPGMA	Protpars	Fitch	NJ	UPGMA	Protpars	Fitch	NJ	UPGMA	

α	77	100	77	94	80	100	100	87	52	100	100	93	71
β	100	100	100	100	100	100	100	100	100	100	100	100	100
γ	94	92	100	58	94	77	100	98	97	91	99	98	95
δ	60	43	100	79	100	100	100	100	54	100	100	100	37
β+γ	52	82	50	99	70	77	82	84	48	95	77	76	64
δ+ξ	100	100	68	15	77	100	38	-	100	49	69	-	72
κ	100	100	100	100	100	100	100	100	100	100	99	99	100
λ	68	100	100	100	88	100	100	100	66	100	100	100	89
μ	95	100	100	100	92	99	100	100	97	95	99	99	97
μ+κ+λ	42	92	97	88	49	99	94	95	33	100	98	89	35

E7	Clustalw				Dialign				Tcoffee				consensus

	Protpars	Fitch	NJ	UPGMA	Protpars	Fitch	NJ	UPGMA	Protpars	Fitch	NJ	UPGMA	

α	54	90	86	99	-	92	84	89	63	85	84	97	30
β	26	96	89	94	99	92	89	84	64	85	96	88	57
γ	58	53	99	96	94	100	83	72	96	94	98	100	81
δ	55	15	-	-	-	47	-	-	-	55	25	-	35
β+γ	-	100	-	-	-	-	-	-	-	-	-	-	-
δ+ξ													-
κ	-	-	-	-	38	-	-	-	88	-	29	-	33
λ	-	95	96	99	99	95	94	96	84	86	91	91	94
μ	100	93	91	99	100	92	94	97	92	96	99	100	92
μ+κ+λ	-	-	-	-	-	-	-	-	-	-	-	-	-

URR	Clustalw				Dialign				Tcoffee				consensus

	dnapars	Fitch	NJ	UPGMA	dnapars	Fitch	NJ	UPGMA	dnapars	Fitch	NJ	UPGMA	
							
		K2/ML	K2/ML	K2/ML		K2/ML	K2/ML	K2/ML		K2/ML	K2/ML	K2/ML	

α	-	-/62	-/-	-/91(1)	99	-/-	np/-	np/-	20	-/15(1)	-/-	89(1)/88(1)	19(1)
β	98	100/100	100/100	100	-	-/-	np/-	np/-	97	-/30	-/-	97/96	51
γ	-	100(2)/100(2)	100(2)/100(2)	100(2)/100(2)	-	-/-	np/-	np/-	-	-/-	-/-	-/-	66(2)
δ	96*	69/62	100	100/100	99	-/-	np/30	np/44	94	85/49	100/99	100/100	80
β+γ	-	-/-	-/-	-/-	-	-/-	np/-	np/-	-	-/-	-/-	-/-	-
δ+ξ	-	-/-	-/-	-/-	-	-/-	np/-	np/-	-	-/-	-/-	-/-	-
κ	-	-/-	-/-	-/-	-	-/-	np/-	np/-	81	-/-	-/-	-/-	-
λ	100	73/-	68	99	-	-/-	np/-	np/-	93	61/49	-/-	98/92	-
μ	-	75/74	58	100/99	-	-/-	np/-	np/-	86	97/98	97/99	100/99	64
μ+κ+λ	-	-/-	-/-	-/-	-	-/-	np/-	np/-	-	-/-	-/-	-/-	-

(1) HPV2 did not cluster together with the rest of the alpha genus

(2) HPV4 did not cluster together with the rest of the gamma genus

np: the high divergence values did not allow the algorithm to rend a solution

Phylogenies were reconstructed with three different alignments, CLUSTALW, DIALIGN and TCOFFEE, subsequently analysed with four different phylogenetic algorithms: a parsimony based algorithm – PROTPARS for protein sequences and DNAPARS for DNA sequences -, and three different matrix-based algorithms: FITCH, Neighbor-Joining (NJ), and UPGMA. Matrices were generated with PROTDIST or DNADIST. For DNA, two different nucleotide substitution models were used, the Kimura-two parameter model (K2) or a maximum-likelihood model (ML). Numbers refer to the percentage of times a given group is recovered in the consensus tree for each reconstruction method, after a bootstrap of 1000 cycles. The column "consensus" gathers the output of the CONSENSE programme with trees from all independent algorithms as input. Some algorithms could not work with the DIALIGN alignment as input, due to the extreme divergence between the sequences. This fact is marked as "np" in the corresponding columns. The support values decrease in the order L1>E7>URR. This reflects the diversity of the evolutionary pressures along the genome of the papillomaviruses. Some of the genera stably recovered according to the L1 protein phylogeny appear with a lower support for the E7 protein phylogeny, and do not appear as definite groups for the URR phylogeny. This is the case for genera alpha, kappa or lambda. Some other genera appear confidently with independence of the element analysed. This is the case for genera beta, gamma and delta. This shows that there are differences in the evolutionary patterns between the members of different clades.

We and others have already shown that the phylogenetic relationships between PV taxa are not homogeneous throughout the whole PV genome [21,24-26]. Instead, they depend on the segment of the genome being considered. The results presented here stress further this concept, as they show that the support for the different individual taxa is different with regards to different elements of the genome. Some taxa are consistently recovered. This is the case of beta PVs, infecting Primates, and delta PVs, infecting Artiodactyla. These taxa appear with good bootstrap values in the consensus tree that gathers twelve phylogenetic reconstructions, and therefore show a homogeneous evolutionary pattern (Table 1).

Alpha PVs have undergone a complex evolutionary history [21], and the picture for the phylogeny of the URR region adds further complexity to it. Alpha PVs do not cluster together according to the URR phylogeny. Only the alignment with DIALIGN analysed with the parsimony approach was able to recover the alpha PV as a group, but even in this case the rendered topology did not resemble that of L1 or that of E7. In the rest of the cases there was no obvious pattern explaining the branching topologies. In all analysed trees, however, as well as in the consensus tree, the alpha PV in species 10 -HPV6, 13, 74, CPV1 and PCPV1- clustered together with a good bootstrap support: 79% support in the consensus tree. This was not the case for other alpha PV species. The most evident cases were HPV16 and HPV2. Thus, HPV16 did not cluster with HPV33 and HPV52 the other two analysed members of the alpha PV species 9. On the other hand, HPV2 did not cluster with the rest of the alpha PVs, and other viruses were unexpectedly closer to the rest of the alpha group than HPV2.

Kappa PVs -infecting Lagomorpha-, lambda PVs -infecting Carnivora- and mu PVs -infecting Primates- appear together regarding the L1 protein sequence, and it has been described that they share an ancient common ancestor [21]. Regarding the E7 protein sequence, lambda and mu PVs appear together, but kappa PVs do not cluster with them. This has been previously interpreted as a consequence of the different evolutionary patterns of the early and late genes in these PVs [21]. The phylogeny of these taxa according to the URR adds further complexity. Species in genus mu -HPV1 and 63- are consistently recovered as a cluster in the URR phylogenetic consensus tree. Genera kappa and lambda, on the contrary, do not appear as separate entities, although the four species analysed here -CRPV, ROPV, COPV1 and FdPV- cluster together with a 39% support in the final consensus tree.

Our results therefore show that the reconstruction of the phylogeny of the URR based exclusively on sequence alignments does not provide stable topologies. Molecular phylogenetic algorithms rely on the basic assumption that species closely related share identities/similarities in their sequences, and that species with high evolutionary distances show more dissimilar sequences. However, certain conditions might result in the recovery of the false tree, such as particular branch-length combinations, heterogeneous evolutionary rates in different branches of the tree or heterotachy in different positions within a sequence [27,28]. The URRs are highly heterogeneous along the different PVs, both in composition and in length. These facts could prevent a proper phylogenetic reconstruction [29]. Since it has been proved that different regions of the genome of the PV show different evolutionary distances, we have analysed this issue in the context of the evolution of the URRs.

### The untranslated regulatory region of the papillomaviruses has diverged faster than the rest of the genome

**Figure 1:**
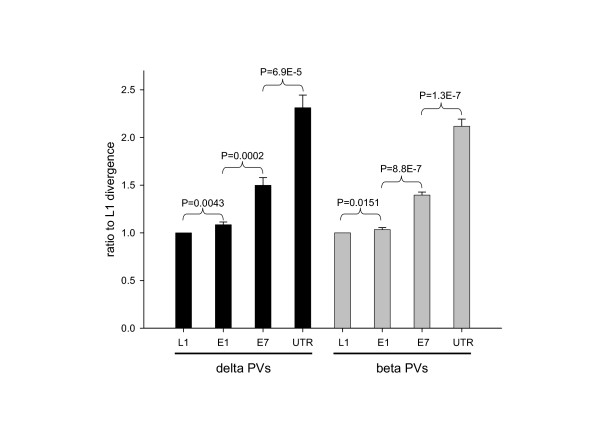
**Relative branch length for the consensus trees of the L1, E1, E7 genes and the URR for the beta and delta papillomaviruses**. Evolutionary distances in substitutions per site from present sequences to the last common ancestor of each genera were calculated on the consensus tree estimated after CLUSTALW alignment, distance matrix construction under the neighbor-joining conditions and a Kimura-two parameter nucleotide substitution model, and bootstrapped 10000 times. The two clades were recovered in all the consensus trees, independently of the analysed element. Distances were normalised for each virus individually, with respect to the L1 distance. *P *values show the results of a two-tailed paired Student's t test for each virus, for the values of the elements connected by the arrow brackets. There is a statistically significant gradient in the divergence distances in the order L1<E1<E7<URR. Divergences in the URR are more than two times higher than in the comparable L1 gene, in both beta and delta clades. The high values of branch-length could account for the low support values obtained for the phylogenetic reconstructions of the URR.

In general, the support values for the different taxa are higher for the L1 protein than for the E7 protein and are lowest for the URR (Table 1). We have already shown that late proteins have diverged less than early proteins in PVs [21,26]. Phylogenetic reconstruction strongly depends on the sequence similarity, and all algorithms tend to fail when sequences are evolutionary too far from each other [27,30]. We have therefore addressed the question whether the poor results in the phylogenetic reconstruction for the URR could be due to a relatively higher divergence rate. Results are shown in Figure 1. We have analysed the phylogeny of the L1, E1, E7 and URR sequences at the DNA level. The analysis was restricted to the genera beta and delta, since these were the only taxa that rendered confident clustering of all their members based on the URR (Table 1). There is an obvious gradient of normalised divergence rate as follows: L1<E1<E7<URR, in both beta and delta PV. The results communicated here for L1, E1 and E7 at the DNA level coincide with previous reports for the corresponding protein sequences [21]. In genera beta and delta, the URR sequences have diverged more than twice as much as the corresponding L1 sequences. These results are not unexpected, due to the lack of coding regions in the URR. However, the phylogenies of genera beta and delta could be properly determined regarding only to the URR sequences, and phylogenetic reconstruction is exclusively sequence-dependent. High divergences hamper proper alignments, and it is not possible to properly infer phylogenetic relationships without good alignments, as exemplified before for the alpha PVs. Thus, despite the absence of coding regions in the URR our results point towards the existence of conserved elements or stretches, which might have a functional importance. All these facts make it obvious that alternative tools are required for the proper analysis of the relationships between PVs, with regards to the URR.

### Different papillomaviruses contain different transcription factor binding sites in the untranslated regulatory region

Our results in the analysis of the phylogeny of the URR in the PVs suggest the existence of locally conserved motifs, which could be embedded in a less conserved environment. It is thought that all PVs contain binding sites in the URR for the E2 protein, as well as for other transcription factors [29]. Although it is believed that most URR contain the same repertoire of TFBS [23,29], experimental confirmation has only been provided for a handful of PVs. We have addressed the question whether the TFBS could be some of the conserved elements in the URR that allowed the phylogenetic reconstruction of certain PV taxa. We have therefore predicted the presence of TFBS in the URR of a representative selection of PVs with the software MATCH. The algorithm compares the sequence to be analysed with a matricial description gathering experimental information about the TFBS sequence. A similar approach was already suggested for HPV31 using the TRANSFAC database [23]. We have chosen a rather generous approach, computing a sum of both error rates – false positive and false negative predictions – to find cut-offs that give an optimal number of false positives and false negatives. The results yielded a list with the presence of the different considered TFBS in the corresponding URR sequences.

**Figure 2:**
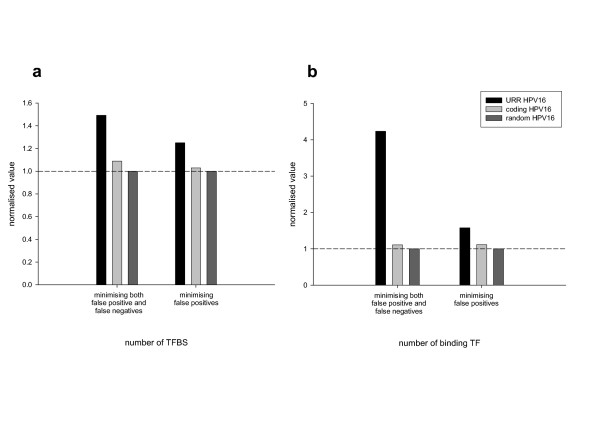
**Density of predicted TFBS and binding TF in different DNA sequences**. Transcription factor binding sites (TFBS) were predicted with MATCH, using the nucleotide matricial description of each site as compiled in TRANSFAC. Predictions were run on three DNA sequences: the URR of HPV16, the HPV16 full-genome except the URR, and a random DNA sequence with the same base composition than HPV16. Results were normalised with respect to the actual predictions in the randomised sequence. Values are shown for the total number of predicted TFBS (a) and for the total number of predicted binding TF (b) since some TF were predicted to have more than one binding site.

The *in silico *prediction of probable TFBS in the URR of PVs with the software MATCH yielded results comparable to the experimental ones. As an example, the URR sequence of HPV16 consisted of 832 bp and was predicted to contain 77 binding sites for 30 different transcription factors. The density of TFBS in the URR was higher than in the rest of the HPV16 genome, and also higher than in a random DNA sequence with the same base composition -32.9%A, 30.6%T, 19.1%G, 17.4%C- (Fig. 2). Furthermore, all the previously experimentally determined TFBS in the URR of HPV16 were recovered as predicted, thus validating our approach. An example of the results for five different PVs from genera alpha, beta and gamma is given in Table 2. From the results shown here it can be inferred that different PVs contain different TFBS in the URR. Some TFBS are predicted to be present in most PVs. This is the case of AP-1, AREB-6, CF2-II, E2, Elf-1, Freac-7, HFH-3, Oct-1, Skn-1 or v-Myb. Other TFBS are restricted to only certain PVs, which could be seen to reflect differential host species- cell type- or differentiation state-specificity. The information thus gained *in silico *might widen our knowledge of the potential reciprocal virus-cell interactions and guide future experimental approaches.

**Table 2:** Example of predicting the presence of transcription factor binding sites in the upstream regulatory region of the papillomaviruses.

	α			β	γ		α			β	γ
			
	HPV16	HPV18	HPV6	HPV8	HPV4		HPV16	HPV18	HPV6	HPV8	HPV4
AbaA	+	+		+	+	HFH-8	+	+	+	+	+
AhR/Arnt	+		+			HLF	+				
AP-1	+	+	+	+	+	HNF-3beta	+		+	+	+
AREB6	+	+	+	+	+	lk-1					+
Arnt	+		+		+	Lmo2 complex		+	+	+	+
Athb-1	+				+	Mat1-Mc	+	+	+	+	
Bcd		+	+	+	+	MATa1				+	
Brc- Z1				+		Max	+				
BR-c Z4	+	+	+	+		MCM1	+				
Brn-2	+		+	+	+	MYB.Ph3	+				+
CCAAT box			+			NF-E2				+	
C/EBP		+		+		NF-Y		+	+	+	+
CDP CR3+HD		+		+		Nkx2-5	+		+		
c-Ets-1(p54)	+	+	+		+	N-Myc	+				
CF2-II	+	+	+	+	+	NRF-2					+
CHOP-C		+	+	+		oct-1	+	+	+	+	+
c-Myb	+	+	+	+	+	OCT-x					+
c-Myc/Max	+					PacC			+		+
Croc	+			+		PHO4	+		+		+
dl		+			+	RAP1		+			
E2	+	+	+	+	+	RFX1		+			
E2F		+				RORalpha1	+		+		
E47		+	+	+	+	S8	+	+	+		+
E74A					+	SBF-1		+	+	+	+
Elf-1	+	+	+	+	+	Skn-1	+	+	+	+	+
Elk-1	+				+	Sn		+	+	+	
ER	+				+	Sox-5	+	+	+	+	+
FOXD3	+		+	+	+	SOX-9	+	+	+	+	+
FOXJ2	+	+	+	+		STATx				+	+
Freac-2	+		+	+		StuAp		+	+		
Freac-7	+	+	+	+	+	Su(H)		+			
GATA-1		+	+	+	+	TATA	+	+	+		+
GATA-2		+	+		+	TCF11				+	
GATA-3		+				TGIF	+				+
GATA-x		+	+	+	+	USF	+		+		
GBP	+		+		+	VBP			+		
GCN4	+		+	+	+	v-ErbA			+		
Gfi-1	+					v-Maf	+				
Hand1/E47				+	+	v-Myb	+	+	+	+	+
Hairy			+			XFD-1	+			+	
HAP2/3/4					+	XFD-2	+		+	+	
HFH-1	+		+	+		YY1	+				
HFH-3	+	+	+	+	+	Zeste				+	+

Transcription factor binding sites were predicted with MATCH, using the nucleotide matricial description of each site as compiled in TRANSFAC. The coincidence levels between the binding site sequence and the sequence in the URR were fixed to optimise simultaneously the number of false positives and false negatives. It is obvious that different papillomaviruses contain different transcription factor binding sites in their URR. Some of them are common to all papillomaviruses, such as AP-1, E2, HFH-3 or Oct-1. Other TFBS are type-exclusive, genus-exclusive, or are shared by papillomaviruses that infect the same host. The high dimensionality of these results makes it necessary to analyse them with information reduction techniques, such as principal component analysis or genetic algorithms.

It might be argued that the *in silico *prediction of TFBS might yield both false positive and false negative results. The higher density of predicted TFBS in the URR as compared to the rest of the genome, as well as the concordance of the predictions with the experimental data suggest a low risk of false negative results. Regarding the putative false positive results, although it is possible that some of the predicted TFBS are really not such, our approach addresses the conservation of functional DNA stretches in similar viruses. As an example, the presence of a HFH-3 binding site in very different viruses highlights the importance of the conservation of this DNA sequence, even if it does not act as a HFH-3 binding site.

### Beta PVs are more homogeneous than alpha PVs regarding the repertoire of predicted TFBS in the URR

**Figure 3:**
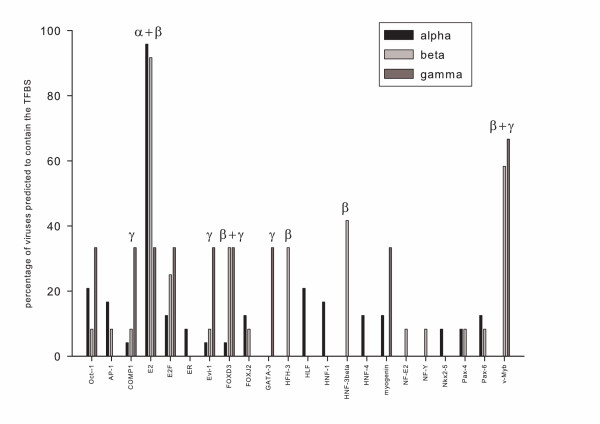
**Presence of predicted transcription factor binding sites in alpha, beta and gamma papillomaviruses**. Transcription factor binding sites were predicted with MATCH, using the nucleotide matricial description of each site as compiled in TRANSFAC. The coincidence levels between the binding site sequence and the sequence in the URR were fixed to minimise the number of false positives. The repertoire of transcription factor binding sites is different in different papillomaviruses. Some binding sites, such as E2, are present in most of the analysed viruses. Others are preferentially present in beta and gamma genera, such as v-Myb or FOXD3, in genus beta, such as HNF-3beta or HFH-3, or in genus gamma, such as COMP1 or GATA-3.

Our results suggest that the repertoire of TFBS is different in different PVs. We have therefore hypothesised that there are patterns of presence/absence of TFBS that could be different in different PVs taxa. To test this hypothesis we predicted again the presence of TFBS in the URR of PVs, with a more conservative approach, aiming to minimise the number of false positives. The URRs were then grouped according to the present PV classification, and the results analysed and searched for conserved patterns of presence/absence of TFBS in the different viral groups. A simple initial display of the results for alpha, beta and gamma PVs confirmed our hypothesis, as shown in Figure 3. It can be seen that certain TFBS are predicted to be present with high confidence in some of these taxa, but not in others. This is the case for v-Myb, present in beta and gamma PVs, or HNF-3beta, present in gamma PVs. Interestingly, the presence of the E2 binding sites in gamma PVs was relatively low, and should be experimentally analysed. The absence of a predicted E2 binding site in gamma PVs is a result of the stringent conditions chosen for the TFBS predicted, since this binding site was also predicted in gamma PVs in the above described les restrictive conditions. However, the selective disappearance of this predicted E2 binding site in particular taxa might also be highly informative. It could reflect slightly different sequence specificity of the E2 protein in these viruses, and/or a different regulatory scheme.

The achieved results consisted of a matrix of ninety-seven predicted TFBS along the sixty-one analysed URR. As shown in Fig. 2, the density of both predicted TFBS and predicted binding TF was higher in the URR than in the rest of the genome or in a random DNA sequence of the same composition. Due to the high dimensionality of the data we approached the hypothesis of the existence of clusters using Principal Component Analysis.

**Figure 4:**
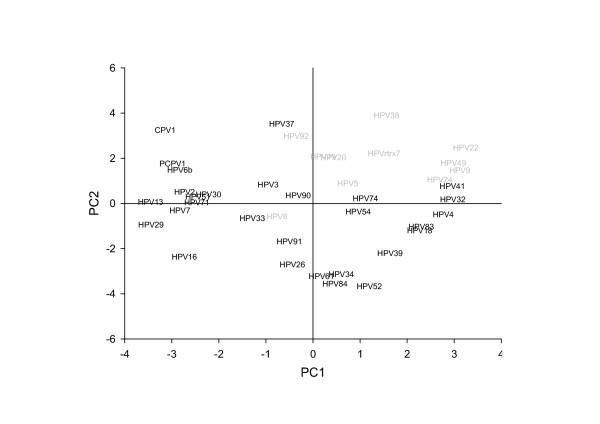
**Principal Component Analysis of the prediction of TFBS in the URR of the papillomaviruses**. The predictions of the presence/absence of TFBS in the URR of the papillomaviruses were analysed by principal component analysis. The figure shows the distribution of alpha and beta papillomaviruses according to the two principal components, PC1 and PC2. Beta papillomaviruses, in grey, cluster together in the upper-right quadrant, whereas alpha papillomaviruses are distributed throughout the other three quadrants. Beta papillomaviruses are therefore more homogeneous according to the principal component analysis of TFBS in the URR than alpha papillomaviruses. This higher diversity in the alpha papillomaviruses is also observed regarding the higher number of species comprised in this genus and to the diversity in their clinical manifestations.

PCA reduces the dimensionality of the initial dataset by finding new variables -components or eigenvectors- which gather the different overall tendencies of the variance in the data [31,32]. The new variables can then be extracted, displayed and analysed. Results for the two more important components in alpha and beta PVs are displayed in Figure 4. It can be seen that beta PVs tend to have high values in both principal components, 1 and 2, and tend to cluster in the upper-right quadrant. On the contrary, alpha PVs do not behave homogeneously and appear dispersed throughout the other three quadrants. Both facts correlate with our findings described above while trying to reconstruct the phylogeny of the corresponding URR: beta PVs clustered together confidently according to the URR sequence, whereas alpha PVs did not appear together as a definite group. In parallel to this, the clinical manifestations associated to infections by beta PVs are more homogeneous than those associated with infections by alpha PVs. All these facts further strengthen the concept of a higher diversity within alpha PVs than within beta PVs [21]. Finally, the newly obtained Principal Components are lineal combinations of the original variables, in our case the presence/absence of TFBS in the URR. Our PCA results suggest that the repertoire of TFBS in alpha and beta PVs is different. We have seeked further confirmation of this statement by analysing the matrix of predicted TFBS by means of genetic algorithms.

### Different taxa within Papillomaviridae show different repertoires of TFBS in their URRs

**Figure 5:**
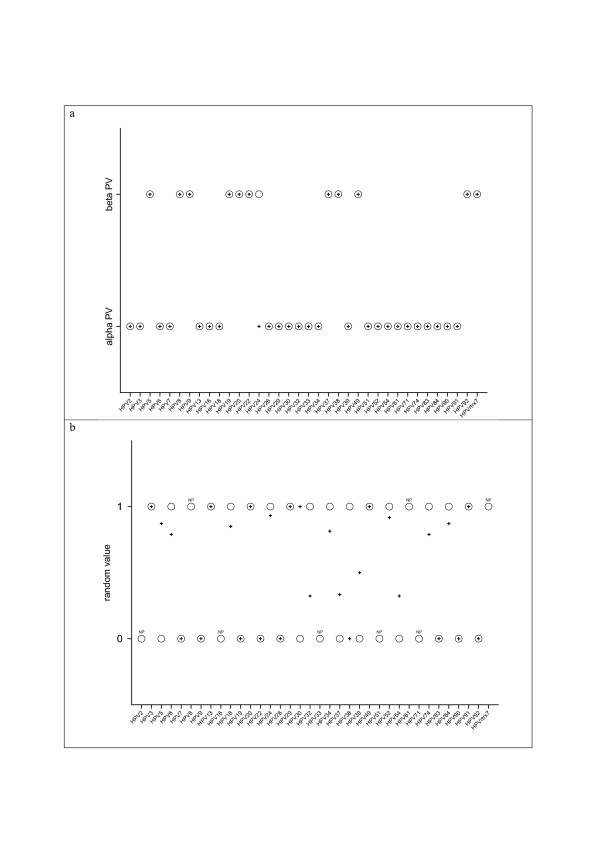
**Predictions by genetic algorithms for the categorisation of alpha and beta papillomaviruses according to the predicted repertoire of TFBS in the URR**. The predictions of TFBS in the URR were analysed by genetic algorithms, including additional information about the genus they belong, alpha or beta (a). Controls were performed with the same predictions, but including random adscription to one of two categories (b). Expected values are shown as open circles, and predicted values are shown as crosses. The elements for which the genetic algorithms could not formulate predictions are labelled as "NP", non-predictable. (a) Genetic algorithms were able to correctly discern alpha and beta papillomaviruses with regards exclusively to their TFBS patterns. (b) The negative control shows that this result is not dependent on the information contained in the prediction matrix itself, but on the categorisation additionally provided. Thus, the repertoire of TFBS in the URR is different between alpha and beta PVs, although both genera infect histologically similar target cells in the same hosts.

Genetic algorithms allow the identification of patterns and the formulation of predictions in highly dimensional datasets [33]. We have applied this tool to the interpretation of our predictions of TBFS in the URR of PVs, using the NEUROSHELLL software. Our aim was to investigate whether different PV taxa could be distinguished attending to the predicted repertoire of TFBS in the URR. Genetic algorithms were trained with the original results, providing additional categorical information. The defined categories corresponded either to actual taxa in the PV classification [2], or to functional criteria, i.e. a given virus infects primates/does not infect primates. We expected therefore from the trained genetic algorithms answers to the questions: i) does a given combination of TFBS distinguish a PV that infect primates from one that infect non-primates? ii) can we predict from the combination of TFBS to which taxon a given PV belongs? As a control, the same input data were randomly classified into the same number of artificial categories. Results are summarised in Table 3. A graphical example for the alpha and beta PVs is provided in Figure 5.

**Table 3:** Predictions by genetic algorithms for the categorisation of different papillomaviruses according to the presence/absence of TFBS in their URR.

input	grouping	correctly predicted	erroneously predicted	unable to predict
all viruses	6 categories, random	44%	66%	0%
	6 categories, taxa: alpha beta gamma+xi kappa+lambda+mu delta+zeta theta+iota	64% (α 85%) (β 84%)	18 (α 7%) (β 8%)	22% (α 7%) (β 8%)
	2 categories, random	61%	39%	-%
	2 categories primate/non primate	90%	2%	8%
human PVs	4 categories, random	30%	56%	14%
	4 categories, taxa: alpha beta gamma mu	76% (α 79%) (β 92%)	12% (α 85) (β 0%)	12% (α 12%) (β 8%)
alpha + beta PVs	2 categories, random	39%	39%	22%
	2 categories, taxa: alpha beta	97%	3%	0%

Genetic algorithms were trained with the matrix describing the presence/absence of TFBS in the URR of the papillomaviruses. An additional column was provided, including either real information about the pertenence of each virus to a genus, or about the nature of the infected host. As a control, additional training was performed on the same matrix providing a column with a random categorisation. Numbers in the table reflect the percentage of viruses for which the genetic algorithms rendered correct predictions, false predictions, or could not formulate any prediction, respectively. Additional values in brackets show the specific accuracies of the predictions for the alpha and beta papillomaviruses. Genetic algorithms were able to discern the pertenence of a given virus to a genus, attending only to the repertoire of TFBS in the URR. Specifically, alpha and beta papillomaviruses were recognised as separate definite clades with high confidence in most of cases. The main targets of both genera are basal cells in the stratified squamous epithelia. These results suggest that alpha and beta papillomaviruses take advantage of different control mechanisms in the same target cell, and/or that they infect different subsets of cells, which are histologically indistiguishable. Finally, genetic algorithms were able to recognise whether a given papillomavirus infects or not primates, regarding only the TFBS patterns in the URR. Papillomaviruses infecting primates are phylogenetically distant, and separated long before the appearance of the primates themselves. Therefore their clustering together according to the repertoire of TFBS suggests a uniformity of regulatory mechanisms, achieved by convergent evolution.

Using the predictions of the presence/absence of TFBS in the URR of different PVs, genetic algorithms were able to accurately predict the adscription of a given virus to a given group. In general, a high number of members in a group resulted in more accurate predictions. This was the case for genera alpha, beta and delta. For other groups, still unrepresented, genetic algorithms were unable to generate predictions. This was the case for kappa, lambda, mu, theta or iota PVs.

Genetic algorithms were able to discern between PVs infecting primates and PVs non-infecting primates (Table 3). The algorithms could not formulate any prediction for only one primate PV, HPV4, a gamma PV. Intriguingly, HPV4 also did not cluster together with the rest of the gamma genus when analysing the phylogeny of the URR. For the rest of primate PVs, including human, chimpanzee, bonobo and macaque PVs, the algorithms formulated accurate predictions. Interestingly this included also mu PVs, which are evolutionary distant from alpha, beta and gamma PVs. The phylogenetic reconstruction of the URR allowed the recovery of beta, gamma and mu PVs as separate taxa, but there was no obvious close topological relationship among them. The last common ancestor of alpha, beta, gamma and mu PVs existed before the emergence of primates as a taxon, possibly even before the major radiative events in the mammalian lineage. Our present results, therefore, point towards the convergent evolution of PVs infecting primates, which is not obvious at the mere sequence level, but is noticeable when analysing the URR at a functional level, i.e. considering the putative TFBS encoded therein.

Genetic algorithms were able to discriminate in virtually all cases between alpha and beta PVs with regards to the TFBS repertoire in the URR (Figure 5). Moreover, genetic algorithms could also correctly identify alpha and beta PVs as definite groups, both when trained with the data corresponding to all analysed PVs, and when trained with the data corresponding to human PVs only (Table 3). These results further show the strength of our approach and suggest interesting implications in the differential regulation caused by both virus genera. On the one hand, we were able to recover alpha PVs as a distinct group, despite the absence of a high degree of sequence similarity among their members. On the other hand, alpha and beta PVs share histologically the same host, namely the keratinocytes in the basal cell layer of stratified squamous epithelia in primates [2,8]. However, the repertoire of TFBS is different in alpha and beta PVs. Different hypotheses could explain this fact. This might reflect the existence of different subpopulations of keratinocytes in the basal cell layer, histologically not distinguishable, with differential susceptibilities to the infection by alpha and beta PVs. Alternatively, alpha and beta PVs could have taken advantage of different protein expression regulatory mechanisms in the infected cell, which would correlate with the presence of different TFBS in their URR.

## Conclusion

The URR of the PVs is at least partially responsible for the tissue tropism, differential transcription, and for the changes in transcription patterns related to host-cell differentiation [8]. The sequence divergence in the URR is very high, and this fact prevents a proper phylogenetic reconstruction of the URR for most of the PV genera. We have therefore addressed the *in silico *analysis of the URR from a functional point of view. We have predicted the presence of TFBS in the URR of a phylogenetically representative selection of PVs. The results were analysed by means of principal component analysis and genetic algorithms.

We have shown that different PVs have different repertoires of TFBS in the URR, even in PVs that infect the same host. This fact could correlate with differential expression patterns and changes as a response to host-cell differentiation. PVs infecting primates show a characteristic TFBS fingerprint, and can therefore be distinguished from other PVs. Since these PVs are polyphyletic [21], the similarities in the functionality of the URR might have appeared as a result of convergent coevolution, arisen under the evolutionary pressure of sharing the same host. The alpha and beta PVs genera are stably recovered as distinct groups according to the TFBS they contain, despite the absence of a high degree of sequence similarity between their members. Both genera infect the same cell type in the same host, but the differences in the TFBS they contain may reflect the existence of different subtypes of keratinocytes and/or the existence of different regulatory mechanisms in different viruses.

Our results indicate that the diversity among alpha PVs regarding the URR is higher than among other groups, such as beta or delta PVs. This suggests that a thorough analysis of the repertoire of TFBS within the alpha PV genus could provide us with functional hints explaining the differences in the biology of their members, such as their differential association with benign or malignant growth.

Finally, it is obvious that different PVs have different repertoires of TFBS. Thus far, most of research concerning URR and transcriptional regulation has focused for obvious reasons on high-risk PVs. Our results again stress the concept that different viruses are different organisms, with potentially different biological properties that might not be directly extrapolated from the results of a human-based biased selection of PVs. The broadening of the number and diversity of the PVs to be empirically studied will surely provide us not only with a broader knowledge of *Papillomaviridae*, but also will strengthen our armoury against the diseases they cause.

## Methods

### DNA sequences. Taxonomic diversity

The PV genome sequences were retrieved either from Los Alamos HPV Sequence Database or from the public databases at EMBL. At present, most of the complete PV sequences belong to alpha, beta and gamma human PVs [2]. To avoid overrepresentation of these taxa, a representative selection of sequences was chosen, adequately covering all the human PV species comprised therein, as follows: alpha-PV HPV32 [NC_001586] (species 1), HPV3 [NC_001588] and 29 [NC_001685] (species 2), HPV61 [NC_001694], 83 [NC_000856] and 84 [NC_002676] (species 3), HPV2 [NC_001352] (species 4), HPV26 [NC_001583] and 51 [NC_001533] (species 5), HPV30 [NC_001585] (species 6), HPV18 [NC_001357] and 39 [NC_001535] (species 7), HPV7 [NC_001595] and 91 [NC_004085] (species 8), HPV16 [NC_001526], 33 [NC_001528] and 52 [NC_001592] (species 9), HPV6 [NC_000904], 13 [NC_001349] and 74 [NC_004501] (species 10), HPV34 [NC_001587] (species 11), HPV54 [NC_001676] (species 13), HPV90 [NC_004104] (species 14) and HPV71 [NC_002644] (species15); beta-PV HPV5 [NC_001531], 8 [NC_001532], 14 [NC_001578], 19 [NC_001581] and 20 [NC_001679] (species 1), HPV9 [NC_001596], 22 [NC_001681], 37 [NC_001687] and 38 [NC_001688] (species 2), HPV49 [NC_001591] (species 3), HPV92 [NC_004500] (species 4), and HPV24 [NC_001683] and HPVRTR [NC_004761] (not assigned); gamma PV HPV4 [NC_001457] (species 1), HPV48 [NC_001690] (species 2) and HPV 60 [NC_001693] (species 4). Other HPV sequences distantly related to the former were also included: HPV1 [NC_001356], 41 [NC_001354] and 63 [NC_001458]. All the non-human PV complete sequences were included, aiming to cover the widest possible interval of host diversity, as follows: PV infecting Primates, *Pan troglodytes *PV [NC_001838] (CPV), *Pan paniscus *PV [NC_006163] (PCPV) and *Macacca mulatta *PV [NC_001678] (RHPV1); PV infecting Rodentia, *Mastomys natalensis *PV [NC_001605] (MnPV); PV infecting Cetartiodactyla, *Phocoena spinipinnis *PV [NC_003348] (PsPV), *Bos taurus *BPV1 [NC_001522] and 4 [NC_004711], *Ovis aries *PV [NC_001789] (OPV1), *Rangifer tarandus *PV [NC_004196] (RPV), *Alces alces alces *PV [NC_001524] (EEPV) and *Odocoileus virginianus *PV [NC_001523] (DPV); PV infecting Perissodactyla, *Equus caballus *EcPV [NC_004194] and EqPV [NC_003748]; PV infecting Sirenia, *Trichechus manatus latirostris *PV [NC_006563] (TmPV); PV infecting Carnivora, *Felis catus *PV [AF377865] (FPV) and *Canis familiaris *PV [NC_001619] (COPV); PV infecting Lagomorpha, *Oryctolagus cuniculus *PV [NC_002232] (ROPV) and *Sylvilagus floridanus *[NC_001541] (CRPV); PV infecting Aves, Passeriformes, *Fringilla coelebs *PV [NC_004068] (FcPV); PV infecting Aves, Psittaciformes, *Psittacus erithacus *PV [NC_003973] (PePV).

### Phylogenetic analysis

ORF sequences identified in the databases as coding L1 or E7, and DNA sequences corresponding to the entire URR were used for phylogenetic inference. The URRs were defined as the DNA sequences lying between the L1 and the E6 ORFs of the PV circular genome. Three alignment algorithms were used: T-COFFEE, which combines information for both global and local homologies [34], CLUSTALW, a progressive alignment algorithm [35], and DIALIGN [36], a local segment alignment algorithm. The results were fed into the PHYLIP programme package for both parsimony and distance matrix evolutionary analysis. DNA phylogeny was estimated by the parsimony method with DNAPARS. Two distance matrices were also generated with DNADIST, under two different nucleotide substitution models, a Kimura-two-parameter model or a maximum likelihood model. Both distance matrices were then analysed with FITCH, which estimates phylogenies from distance matrix data under the "additive tree model" according to which the distances are expected to equal the sums of branch lengths between the species, using the Fitch-Margoliash criterion. Alternatively, the distance matrix was analysed with NEIGHBOR, under both the Neighbor-Joining and UPGMA methods of clustering. The statistical support was assessed by 1000 cycles bootstrapping with the SEQBOOT and CONSENSE programmes. Thus, three different alignments were analysed with four different phylogenetic methods, yielding twenty-one different estimates of the phylogenetic relationship within DNA PV sequences.

### Prediction of transcription factor binding sites

Transcription factor binding sites (TFBS) in the genome region between the L1 and the E6 genes were predicted with MATCH, designed for searching potential binding sites for TFBS nucleotide sequences. MATCH uses a library of mononucleotide weight matrices from TRANSFAC 6.0. In brief, the MATCH algorithm looks for matches between the nucleotide weight matrices that experimentally define the TFBS and the analysed URR sequence. A matrix similarity value is then calculated depending on the quality of the match between the core sequence of the matrix -the most conserved positions- and a part of the input sequence. A positive match must have a score higher than or equal to the core similarity cut-off. We have chosen a high cut-off value, designed to reduce the number of random sites found by MATCH.

### Analysis of transcription factor binding sites

The results of the prediction of TFBS in the URR were used to build a matrix presence/absence of ninety-two different TFBS in sixty-one different Pvs. Due to the high dimensionality of the matrix it was analysed by Principal Component Analysis (PCA) or by means of genetic algorithms.

PCA is a method widely used in pattern-recognition studies [31,32]. This statistical tool promotes dimension reduction and modelling of the original data by creating new coordinate axes defined according to the principal components (PCs) extracted from the original data. The initial dataset is displayed in an *n*-dimensional space, *n *being the number of defined variables. In our case *n *is the total number of different predicted TFBS in the whole sequence dataset. The first PC is determined by looking for the direction of the maximum residual variance in the *n*-dimensional space. From the remaining data variance -after the removal of the first PC- a second PC that is completely uncorrelated with – i.e. orthogonal to – the first one is extracted, and accounts for the maximum possible remaining dataset variance. PC1 always explains more of the total information than PC2, PC3 and others. The procedure is then repeated until all PCs are generated.

PCA is an extremely useful tool, which maps samples through scores and loadings. Score plots allow sample identification, clarifying whether they are similar or dissimilar, typical or outliers. Moreover, loadings plots allow the checking of the correlation between variables and also enable the variables that contribute most to each principal component to be defined.

A genetic algorithm is a random, yet directed search for an optimal solution to a problem [33]. In our case the problem is the categorisation of the MATCH results describing the presence/absence of the different TFBS in the original URR sequences dataset. We have used the NEUROSHELL software. The algorithm searches for optimisation by first encoding the initial information into a "genetic" formalism. Thus, a population of "organisms" that contain a "genome" made up of "genes" is first formally defined. The genes are the parameters to be optimised and the organisms are solutions to the optimisation problems. In each generation organisms are allowed to recombine and mutate. For each new organism it is calculated how well its parameters solve our categorisation problem. The better they solve the problem the "fitter" they are, and therefore the probability of "surviving" and "breeding" will also be higher. After many generations the error surface is thoroughly explored and the population evolves towards a fitter state. At this point we are provided with an algorithm that weights the contribution of each variable, in our case presence/absence of TFBS, to the solution of the problem, in our case the categorisation of the initial URR sequences. The accuracy of the categorisation predictions can then be tested and their usefulness evaluated.

## Authors' contributions

SGV carried out the Principal Component Analysis and participated in the global data analysis. JRIR participated in the Genetic Algorithm Analysis. AA participated in the design of the study and in the draft of the paper. IGB conceived the study and coordinated it, and performed the TFBS prediction and analysis.
